# Polygenic risk scores: a biased prediction?

**DOI:** 10.1186/s13073-018-0610-x

**Published:** 2018-12-27

**Authors:** Francisco M. De La Vega, Carlos D. Bustamante

**Affiliations:** 1 Department of Biomedical Data Science, Stanford University School of Medicine, Campus Drive, Stanford, CA 94305 USA; 2 Fabric Genomics Inc., Telegraph Avenue, Oakland, CA 94612 USA; 3 Department of Genetics, Stanford University School of Medicine, Campus Drive, Stanford, CA 94305 USA; 4 Chan Zuckerberg Biohub, Illinois Street, San Francisco, CA 94158 USA

## Abstract

A new study highlights the biases and inaccuracies of polygenic risk scores (PRS) when predicting disease risk in individuals from populations other than those used in their derivation. The design bias of workhorse tools used for research, particularly genotyping arrays, contributes to these distortions. To avoid further inequities in health outcomes, the inclusion of diverse populations in research, unbiased genotyping, and methods of bias reduction in PRS are critical.

## Resurgence of polygenic risk scores

There is a renewed interest in developing and applying polygenic risk scores (PRS) to predict the genetic liability of human traits, including predisposition to common diseases [1]. This resurgence is fueled by several major developments: (i) thousands of reports of genome-wide association studies (GWAS) encompassing larger samples, with some studies reaching up to a million subjects [2]; (ii) new methodology for developing PRS from raw GWAS genotypes without relying solely on genome-wide significant hits [3]; and (iii) the availability of large longitudinal cohorts providing the rich phenotype and genetic data [4] needed to validate and test PRS. Validation is needed to prove that a PRS does not overfit the training data, producing inflated results, and requires a sample that is entirely separate from the training dataset to evaluate their performance.

GWAS have been successful in identifying a subset of the genes and causal variants behind polygenic common diseases, such as coronary artery disease (CAD), cancers, and type 2 diabetes. It was initially hoped that once the genetic architecture of a trait was identified, the observed effects of the risk-associated alleles could be used to construct a combined score and to predict individuals at the tail ends of the risk distribution. In the early days of GWAS, the observed effects of the risk alleles were often found to be small, so more GWAS samples were aggregated to achieve greater power and more associated alleles were found, but with even smaller effects. Even when these were accounted for, only a small fraction of heritability seemed to be explained (the so-called ‘missing heritability problem’ [5]), suggesting that the hope of genetic risk prediction would never be realized.

However, new methodologies that relinquished the goal of finding the complete catalog of causal genes, and instead aggregated data from a larger fraction of the genotyped variants that scored below the genome-wide significance threshold, were devised to account for undiscovered loci [6]. These approaches explained a much larger fraction of trait heritability. With larger GWAS and the advent of datasets such as the UK Biobank [4], which collected deep genetic and phenotypic data from approximately 500,000 individuals, the prospect of utilizing PRS as a clinical tool is gaining traction [1].

## Defining the role of PRS in healthcare

The causation of common human diseases is complex as it results from a combination of genetic and environmental factors. A key mission of genomic medicine is to predict the genetic liability of disease on the basis of an individual’s genotype. Identifying those in the population who are at greater risk of disease can result in breakthroughs in healthcare management and can lower costs by reducing unnecessary disease burden and by introducing preemptive therapies or lifestyle changes for those at greater risk. Khera et al. [7] provide an example of how a convergence of factors is starting to realize this mission. PRS constructed from large-scale GWAS of five common diseases could identify individuals within the UK Biobank with high disease risk. The PRS for CAD, for example, found 8% of individuals in the test dataset who exhibited a threefold or more increase in risk for the disease, a fraction of the population that is 20-fold larger than that comprised of individuals carrying monogenic mutations that confer a comparable increase in disease risk. This finding suggests that if this PRS was applied in clinical care, individuals in the > 95% percentile of the CAD risk distribution could be started on statins and prescribed a healthier diet, probably preventing morbidity and untimely mortality in this population.

Many more recent or upcoming studies have used similar approaches to describe PRS for a multitude of traits. And as obtaining genotype array data is becoming more inexpensive, there are now suggestions that the time has come to apply PRS in clinical care [1, 7]. But, are PRS ready for prime time?

## The bias in the machine

There are several potential pitfalls in the construction of PRS that could affect how they perform in real-world clinical populations. One of the most obvious is that they suffer from the same bias that most genetics research experiences: a lack of diversity in the populations recruited for genetic studies [8]. Until recently, over 80% of participants in genetic studies have been of European descent, 14% have been Asian, and just 6% have been from other populations [8]. Disease-associated alleles can have significantly different frequencies between populations as the result of demographic events, such as migrations and population bottlenecks, which can lead to discovery bias. In addition, linkage disequilibrium-based pruning or adjustments performed as part of the construction of the PRS [3] can contribute bias, because of the limited reference haplotype panels for diverse populations. Accordingly, Martin et al. [9] reported that PRS derived from European-based GWAS show biases in different, often unpredictable, directions when tested in non-European cohorts.

A recent report from Kim et al. [10] not only confirms that PRS derived from GWAS of European-ancestry samples can misestimate risk when applied to other populations, but also that the very tools used to genotype the GWAS samples contain bias and contribute significantly to the misestimation of disease risk across populations. These researchers first showed that disease allele frequencies for loci in the National Human Genome Research Institute (NHGRI) catalog of published GWAS studies differ significantly between Europeans and other populations sampled in the 1000 Genomes Project. Second, they observed that Africans exhibit significantly higher risk allele frequencies, a difference that is higher for ancestral risk alleles (i.e., the allele sequence present in hominid common ancestors) than for derived risk alleles (i.e., sequences that arose in the human population more recently). When risk alleles are binned into disease categories, those diseases with a higher proportion of causal ancestral alleles show elevated average risk allele frequencies in Africa. This skew in risk allele frequencies is sometimes discordant with known differences in disease prevalence between populations (e.g., for cardiovascular disease, African-Americans have a higher incidence but a PRS showed lower risk for Africans), implying that genetic disease risks may be misestimated, most significantly for individuals with African ancestry.

Furthermore, the commercial single nucleotide polymorphism (SNP) genotyping arrays used in GWAS have a strong ascertainment bias, as these SNPs were selected from the sequencing data of a small sample of individuals, mostly of European descent. Through simulations, Kim et al. [10] show that this ascertainment bias alone can cause disease risks to be misestimated. On the other hand, simulations using whole-genome sequencing show much reduced (although not completely eliminated) biases in allele frequency differences between Africans and non-Africans, particularly when sample sizes increase. These results suggest that performing GWAS in more diverse samples, which include participants from around the world, is not sufficient to reduce discovery bias [8], because performing such studies with standard commercial SNP arrays would still result in biases. This is an important insight, as SNP arrays are inexpensive and genetic studies planned around the world are cost-constrained. Performing whole-genome sequencing in place of using SNP arrays would alleviate the ascertainment bias problem, but would increase costs by orders of magnitude. How might we resolve this dilemma?

## Overcoming biases

A number of approaches have been proposed to reduce the biases in PRS with respect to their application in populations with diverse or admixed ancestry. Clearly, the inclusion of more diverse populations in GWAS and biobanking is essential to reducing biases and addressing health disparities [8]. These studies also require improved arrays designed for cosmopolitan samples and informed by diverse variant discovery efforts. Whole-genome sequencing would be the ideal platform on which to perform such studies but, until costs drop further, alternative approaches, such as low-coverage sequencing, have been proposed. Low-coverage sequencing at < 1× depth now has costs approaching those of SNP microarrays and could impute a set of genotypes with high accuracy. Imputation relies, however, on haplotype reference panels that are mostly available for individuals of European descent and East Asians, and consequently imputation into other populations is less accurate. In the absence of truly cosmopolitan GWAS data and validation cohorts, statistical adjustments of the PRS derived from European data could be applied to predict risk in other populations more closely. Kim et al. [10] suggest a method that considers whether the risk allele is ancestral or derived and show encouraging results in their simulations, but more research is needed in this area.

## Towards precision health equity

Biases in genetic research have created the potential for health disparities [8]. PRS based on GWAS of European-descent cohorts could become useful in improving health outcomes for individuals from these populations, but currently may misestimate risk in admixed individuals and those of different ancestries [10]. To strive towards health equity in precision medicine and to prevent further health disparities, both study designs that include population diversity and methods to compensate for the biases incurred in constructing PRS need to be prioritized. For the sake of simplicity, we have not discussed important non-genetic sources of health disparities, including discrimination, lack of access to healthcare, and gene-by-environment interactions, which further complicate the problem at hand. Nonetheless, we remain optimistic that a concerted effort to both broaden representation in discovery cohorts and to develop tools to translate these discoveries into actionable healthcare management strategies are the way forward to improving health outcomes for all.

## Abbreviations

CAD: Coronary artery disease
GWAS: Genome-wide association studies
PRS: Polygenic risk score(s)
SNP: Single nucleotide polymorphism

## References

[1] Warren M (2018). The power of many. Nature.

[2] Evangelou E, Warren HR, Mosen-Ansorena D, Mifsud B, Pazoki R, Gao H (2018). Genetic analysis of over 1 million people identifies 535 new loci associated with blood pressure traits. Nat Genet.

[3] Vilhjálmsson BJ, Yang J, Finucane HK, Gusev A, Lindstrom S, Genovese G (2015). Modeling linkage disequilibrium increases accuracy of polygenic risk scores. Am J Hum Genet.

[4] Bycroft C, Freeman C, Petkova D, Band G, Elliott LT, Sharp K (2018). The UK biobank resource with deep phenotyping and genomic data. Nature.

[5] Manolio TA, Collins FS, Cox NJ, Goldstein DB, Hindorff LA, Hunter DJ (2009). Finding the missing heritability of complex diseases. Nature.

[6] Evans DM, Visscher PM, Wray NR (2009). Harnessing the information contained within genome-wide association studies to improve individual prediction of complex disease risk. Hum Mol Genet.

[7] Khera AV, Chaffin M, Aragam KG, Haas ME, Roselli C, Choi SH (2018). Genome-wide polygenic scores for common diseases identify individuals with risk equivalent to monogenic mutations. Nat Genet.

[8] Popejoy AB, Fullerton SM (2016). Genomics is failing on diversity. Nature.

[9] Martin AR, Gignoux CR, Walters RK, Wojcik GL, Neale BM, Gravel S (2017). Human demographic history impacts genetic risk prediction across diverse populations. Am J Hum Genet.

[10] Kim MS, Patel KP, Teng AK, Berens AJ, Lachance J (2018). Genetic disease risks can be misestimated across global populations. Genome Biol.

